# Systematic Review and Meta-Analysis: Prevalence of Meibomian Gland Dysfunction Among Adults Aged 40 Years and Older

**DOI:** 10.3390/jcm15083034

**Published:** 2026-04-16

**Authors:** Balzhan Karimberdiyeva, Zhanat Sadibekova, Aizhan Raushanova, Indira Karibayeva, Nuray Yerbol, Zinat Abdrakhmanova

**Affiliations:** 1 Department of Public Health, Kazakhstan Medical University “KSPH”, Almaty 050060, Kazakhstan; 2 Department of Public Health and Healthcare, South Kazakhstan Medical Academy, Al-Farabi Square 1/1, Shymkent 160019, Kazakhstan; 3 Department of Epidemiology, Biostatistics and Evidence-Based Medicine, Al-Farabi Kazakh National University, Almaty 050040, Kazakhstan; nurai.ilyasova5002@gmail.com (N.Y.);; 4 Department of Health Policy and Community Health, Jiann-Ping Hsu College of Public Health, Georgia Southern University, Statesboro, GA 30460, USA; ik01379@georgiasouthern.edu; 5 Department of Research Management, JSC Research Institute of Cardiology and Internal Diseases, Almaty 050012, Kazakhstan; 6 Department of Public Health, Kazakh-Russian Medical University, Almaty 050000, Kazakhstan

**Keywords:** meibomian gland dysfunction, dry eye disease, prevalence, epidemiology, meta-analysis, older adults

## Abstract

**Background**: Meibomian gland dysfunction (MGD) is a major contributor to evaporative dry eye disease, yet reported prevalence estimates vary widely across studies, largely due to differences in diagnostic criteria, study populations, and settings. We conducted a systematic review and meta-analysis to synthesize available evidence on the prevalence of MGD in adults aged ≥40 years and to examine sources of heterogeneity across studies. **Methods**: We systematically searched PubMed, Web of Science, Scopus, and Google Scholar from inception to November 2025 for observational studies reporting the prevalence of MGD in adult populations. Random-effects meta-analysis of proportions was performed. Subgroup analyses were conducted by study setting and diagnostic approach. Between-study heterogeneity was quantified using the I^2^ statistic. **Results**: Eight observational studies from Singapore, Spain, Russia, Iran, China, Japan, and New Zealand, comprising 20,518 participants, met inclusion criteria. Reported prevalence of MGD varied substantially across studies. The pooled prevalence estimate was 87.08% (95% CI: 65.32–96.02%); however, heterogeneity was extreme (I^2^ > 99%), indicating substantial variability across populations and study methods. This pooled estimate should be interpreted with caution and not as a single representative global prevalence. Studies using clinical signs alone tended to report higher prevalence than those incorporating both signs and symptoms. Differences in study setting and diagnostic definitions accounted for a significant proportion of heterogeneity. **Conclusions**: MGD appears to be commonly detected in adults aged ≥40 years; however, prevalence estimates vary markedly depending on diagnostic criteria and study design. Given the extreme heterogeneity, pooled prevalence estimates should be interpreted with caution and not as a single global prevalence value. Standardized diagnostic definitions and population-based studies using harmonized methodologies are needed to generate more comparable and clinically interpretable estimates of MGD burden.

## 1. Introduction

Meibomian gland dysfunction (MGD) has emerged as a critical public health concern and is now recognized as the leading cause of evaporative dry eye disease (DED), contributing substantially to the loss of ocular surface homeostasis [1]. MGD is among the most prevalent ophthalmic diseases: a global meta-analysis reports an overall prevalence of about 35.8% among the adult population worldwide, with individual study estimates ranging from approximately 3.5% to 70% across different populations [2]. In the United States, the prevalence of MGD is estimated at 21.2% (based on 19,648 participants) [3]; in Africa, hospital-based data show an average of 45.9% (95% CI 27.6–64.1; 4963 patients) [4]. Population-based studies in individual countries report 26.3% in Iran [5], (32.9% in Japan (Hirado-Takushima) [6], 56.3% among Malays in Singapore [7], and 52.6% in Russia (individuals aged ≥40 years) [8]. In clinical and specialized samples, the prevalence is even higher: up to 47–75% among patients preparing for cataract surgery in Japan [9], about 48–58% in Indian and African clinics [10], 54.3% among 6525 patients attending routine ophthalmology visits in 11 countries, and more than 90% among patients presenting with dry eye complaints in specialized clinics in Norway and Taiwan [11].

Meibomian glands are specialized sebaceous glands embedded in the tarsal plates of the eyelids, whose primary function is the production and secretion of meibum—a complex mixture of lipids and proteins that forms the outermost layer of the tear film and is essential for maintaining ocular surface homeostasis. The lipid components of meibum, including wax esters, cholesteryl esters, and triacylglycerols, prevent rapid evaporation of the aqueous tear layer, reduce surface tension, and provide a smooth optical surface [12]. In addition to lipids, meibomian glands secrete a variety of proteins that contribute to the innate immune defense of the ocular surface and modulate inflammatory processes [13]. The integrity of meibomian gland function depends on coordinated programs of meibocyte differentiation, lipid metabolism, and epithelial renewal, which are regulated by epigenetic mechanisms [14].

Meibomian gland dysfunction represents a chronic, progressive disease characterized by terminal duct obstruction and qualitative or quantitative alterations in meibum secretion, which fundamentally disrupts the tear film lipid layer and initiates a cascade of pathophysiological events [15]. When MGD remains undiagnosed and untreated, the natural course of disease progression leads to permanent structural changes in the meibomian glands, including glandular atrophy, ductal dilation, cystic degeneration, and ultimately irreversible meibomian gland dropout [16,17]. The pathogenesis follows a self-perpetuating vicious circle wherein gland obstruction leads to meibum stasis, bacterial proliferation with release of lipases and esterases, increased meibum melting temperature, further obstruction, and progressive gland loss [18]. While recent evidence suggests that partial recovery of gland structure may be possible with early intervention, the prevailing consensus indicates that meibomian gland loss is only partially reversible once significant atrophy has occurred, making early detection and treatment essential to prevent permanent glandular damage [19].

Global epidemiological data indicate that MGD is highly prevalent across diverse populations. A comprehensive meta-analysis of worldwide studies reported a pooled global prevalence of approximately 35.8%, with substantial variation across ethnic groups—ranging from 21% in Africans and Caucasians to as high as 67–71% in Hispanics and Arabs [20]. In the United States, pooled prevalence estimates from recent systematic reviews suggest an MGD prevalence of approximately 21%, though individual studies report rates ranging from 10% to 55% depending on study design, diagnostic criteria, and population characteristics [21]. In East Asian populations, MGD prevalence ranges from 33% to 50%, rising to 51.8–60.8% in individuals over 65 years of age. In an Iranian population-based study, MGD prevalence was reported as 26.3% in adults, increasing dramatically to 71.2% in those over 60 years of age [22]. In Russia, a large population-based study (the Ural Eye and Medical Study) among adults aged 40 years and older demonstrated exceptionally high MGD prevalence rates, with the burden increasing markedly with age [23]. Importantly, the risk of developing MGD increases substantially with advancing age, with prevalence estimates suggesting that up to 70% of Americans over 60 years may have MGD [24]. Men appear to be at higher risk than women, with a pooled odds ratio of 1.24, although the impact of sex on MGD remains equivocal in some studies [11]. Age-related changes in meibomian glands include decreased meibocyte differentiation, reduced cell proliferation, acinar atrophy, gland dropout, and altered meibum composition, all of which contribute to the development of age-related hyposecretory MGD [25].

The burden of MGD extends well beyond ocular discomfort and significantly impacts visual function, quality of life, and economic productivity. Studies demonstrate that individuals with severe MGD experience clinically significant reductions in best-corrected visual acuity (a decline of more than five Early Treatment Diabetic Retinopathy Study (ETDRS) letters) [26]. These functional impairments translate into substantial difficulties with daily activities such as reading, driving, computer use, and outdoor activities, with patients reporting increased time spent on eye care and elevated psychological distress [27]. The impact on workplace productivity is particularly notable, with studies indicating up to a 30% reduction in work productivity among affected individuals. The economic and humanistic burden of MGD-associated DED is considerable, imposing substantial direct costs (medical consultations, medications, procedures) and indirect costs (reduced productivity, absenteeism, diminished quality of life), with burden increasing markedly with disease severity [28].

The prevalence of Meibomian gland dysfunction (MGD) among individuals aged 40 years and older is of fundamental importance, as MGD becomes a widespread condition after this age. Population-based studies show that the proportion of individuals aged 40–80 years with signs of MGD reaches approximately 56% in Singapore and about 30% asymptomatic + 8–9% symptomatic in Spain. In older adults (aged 60 years and above), the prevalence increases to around 70% or higher, leading to an increase in evaporative dry eye syndrome, decreased tear film stability, and deterioration of vision-related quality of life. Moreover, MGD is associated with systemic risk factors such as dyslipidemia, diabetes mellitus, and cardiovascular diseases, which enhance the clinical and social significance of the condition and justify the need for early detection and targeted management strategies in this age group [29,30,31,32,33].

Despite the expanding body of literature on MGD, epidemiological data on its prevalence among adults over 40 years of age remain fragmented and inconsistent. Available studies differ substantially in design (population-based versus clinic-based samples), diagnostic criteria, age ranges, and geographic settings [34]. This heterogeneity complicates the assessment of disease burden and hampers the planning of screening and prevention strategies in high-risk groups [35]. Notably, MGD may remain asymptomatic in a substantial proportion of adults, increasing the likelihood of underdiagnosis in older populations [36]. Furthermore, the absence of standardized diagnostic approaches for MGD—ranging from slit-lamp biomicroscopy and meibography to symptom questionnaires—contributes to the wide variability in reported prevalence estimates [37]. The progressive nature of age-related meibomian gland changes, including acinar atrophy and gland dropout, underscores the necessity of obtaining reliable age-specific prevalence data to inform clinical practice and public health policy [26].

Objectives: Given the high burden of MGD, the critical role of age as a risk factor, and the notable gaps in age-specific epidemiological data across different populations, a systematic review focusing specifically on the prevalence of MGD in individuals over 40 years of age is warranted. The objectives of this systematic review are:

1. To systematically identify and synthesize available observational evidence on the prevalence of meibomian gland dysfunction among adults aged 40 years and older across diverse geographic regions;
2. To estimate the pooled prevalence of MGD in this age group using random-effects meta-analysis;
3. To explore sources of heterogeneity in MGD prevalence estimates through subgroup analyses and meta-regression, examining the influence of diagnostic methods, population characteristics, and study design.

## 2. Materials and Methods

This systematic review was conducted following the Preferred Reporting Items for Systematic Reviews and Meta-Analyses 2020 (PRISMA 2020) guidelines [38]. The review protocol was submitted to PROSPERO (ID: CRD420251245395) after confirming that no similar reviews existed.

### 2.1. Search Strategy

The following databases were searched: PubMed, Scopus, Web of Science, ScienceDirect, and Google Scholar. Filters applied included publication in English, publication in scholarly journals, and document types limited to articles, research articles, and early access articles. No restrictions were placed on the year of publication.

To define the search terms, a preliminary PubMed search was conducted to identify relevant keywords from the titles and abstracts of studies focusing on the prevalence of meibomian gland dysfunction. Based on this preliminary search, the following keywords were used in the final search strategy: “Meibomian gland dysfunction [MeSH]” OR “MGD” AND “Prevalence” OR “Incidence” OR “Epidemiology [MeSH]”. Further details on the search strategy are provided in Table 1.

### 2.2. Eligibility Criteria, Study Selection and Data Collection

Table 2 presents the eligibility criteria used to select articles in accordance with the Population, Exposure, Comparator, Outcome, and Study Design (PECOS) framework for this review on the prevalence of MGD. The population of interest included the general adult population aged 40 years and older; studies conducted exclusively among children, adolescents, or adults younger than 40 years were excluded. Studies focusing solely on highly specific subgroups (e.g., animal models or exclusively experimental cell or tissue samples) were also excluded. The exposure criteria encompassed clinically diagnosed MGD based on slit-lamp biomicroscopy, meibography, and/or standardized symptom questionnaires. Studies that reported only dry eye disease without separate MGD data, or that focused on treatment interventions (such as devices or pharmaceuticals) without providing baseline prevalence, were excluded. No comparator was applicable. The primary outcome of interest was the prevalence of MGD, defined as the number of individuals diagnosed with MGD out of the total number of participants assessed. Studies that examined only toxicity, safety, or efficacy of specific treatments, without reporting MGD prevalence in a defined adult population, were excluded. The review included cross-sectional and other observational (population-based or clinic-based) studies, whereas randomized controlled trials, narrative reviews, systematic reviews, editorials, commentaries, conference abstracts, and articles published in languages other than English were excluded.

The eligibility assessment and data collection were conducted in accordance with PRISMA guidelines. Two independent researchers (Z.A. and I.K.) performed a standardized search across all selected databases. After completion of the searches, all records were exported and combined in Zotero Citation Manager version 7.0.32. [39], where duplicate entries were identified and removed. Only unique records were then screened for relevance based on titles and abstracts. In the final stage of eligibility assessment, full-text articles were evaluated against the predefined inclusion and exclusion criteria, and relevant data were extracted using a standardized data collection form. Extracted information included the first author’s last name, year of publication, country, study design, condition assessment setting (general population or clinical), age range or mean age, total population assessed, number of participants with MGD, diagnostic method used, and proportion of female participants. Two independent datasheets were then compared and merged. Any discrepancies in study selection or data extraction between the two reviewers (Z.A. and I.K.) were resolved through discussion and evaluation with a third author (B.K.), and consensus was reached for all studies included in the review.

### 2.3. Meta-Analysis

The statistical synthesis of proportional data was performed in R version 4.5.1 (13 June 2025) [40] within the RStudio environment (version 2025.9.0.387). Implementation relied on two specialized R packages—meta and metafor—for aggregating prevalence estimates across studies. Using a random-effects modeling approach, we synthesized the pooled prevalence estimates of MGD with corresponding 95% confidence intervals (CI) using logit transformation. Forest plot visualizations were employed to present the analytical findings. Assessment of between-study variability was accomplished through calculation of the I^2^ index. To identify and understand sources of variation across studies, we performed subgroup stratification by research context (community-based versus hospital-based settings). Additionally, sensitivity evaluations and iterative exclusion procedures were implemented. Publication bias assessment was not performed, as the number of included studies in the meta-analysis was less than 10.

### 2.4. Risk of Bias

Quality assessment was performed using a modified version of the Newcastle-Ottawa Scale (NOS) adapted for observational cross-sectional designs. The adapted instrument comprised six evaluation domains distributed across three categories: participant selection (three items), study comparability (one item), and outcome assessment (two items). Scoring permitted a maximum of one point per domain, with the comparability category allowing up to two points, yielding a possible range of 0–7 points. Higher numerical values reflected superior methodological quality. Two independent reviewers (Z.A. and I.K.) conducted parallel quality assessments following protocol standardization. Agreement between raters was quantified by a third investigator (B.K.). Studies achieving a score ≥ 5 points were classified as adequate quality and retained for the systematic synthesis.

### 2.5. Certainty of Evidence Evaluation

Evidence grading followed recommendations established in the Cochrane systematic review handbook. The GRADE methodology, implemented in RStudio, was applied to evaluate confidence in findings across five evaluation domains: methodological limitations (using the NOS framework), consistency of effect estimates (via I^2^ values), applicability to the target population (based on PICO framework alignment), precision of estimates (determined by whether 95% CIs encompassed predefined clinical benchmarks), and publication bias was not formally assessed due to the limited number of included studies (*n* = 8), as statistical tests for funnel plot asymmetry require a minimum of approximately 10 studies to have adequate power.

## 3. Results

### 3.1. Study Selection and Characteristics of the Included Studies

**Figure 1:**
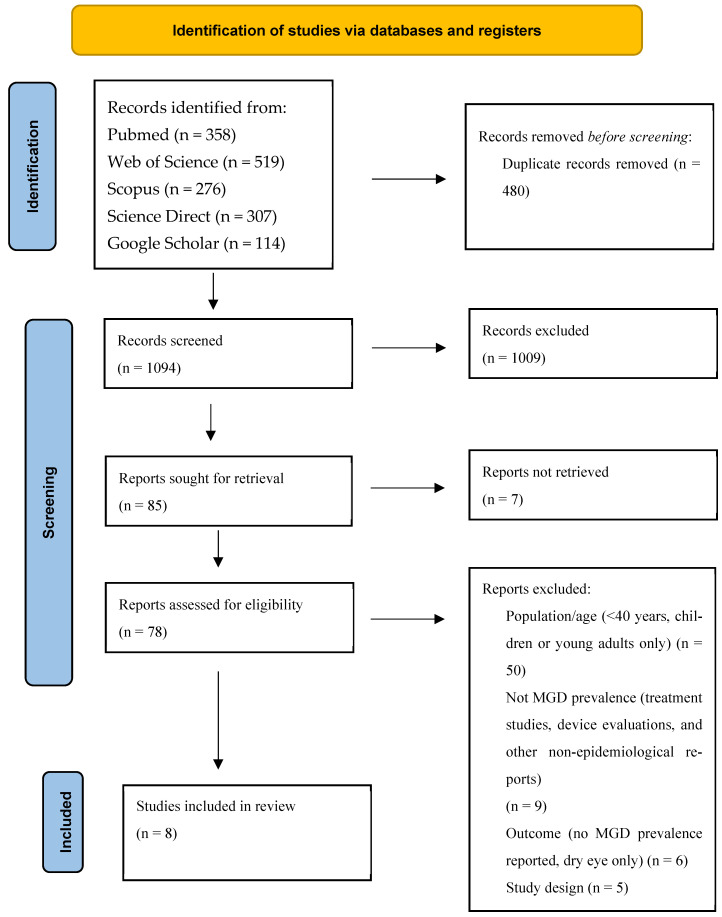
PRISMA 2020 flow diagram for new systematic reviews which included searches of databases.

According to the PRISMA flow diagram, a total of 1574 records were identified across PubMed, Web of Science, Scopus, ScienceDirect, and Google Scholar. After removing 480 duplicates, 1094 unique titles and abstracts were screened for eligibility. Of these, 1009 records were excluded at the screening stage as clearly not relevant to the review question. Full texts were sought for 85 articles, of which 7 could not be retrieved despite attempts to obtain them. Seventy-nine full-text articles were assessed for eligibility, and 70 were excluded for the following reasons: wrong population or age (participants younger than 40 years, pediatric or adolescent samples only; *n* = 50), not MGD prevalence (treatment studies, device evaluations, or other non-epidemiological reports; *n* = 9), wrong outcome (no MGD prevalence reported, dry eye only; *n* = 6 [41,42,43,44,45]) and wrong design (non-observational designs such as trials or reviews; *n* = 5) [44,45,46,47,48]. Ultimately, 8 observational studies fulfilled all PECOS eligibility criteria and were included in the systematic review and quantitative synthesis. The PRISMA flowchart summarizing the study selection and inclusion process is presented in Figure 1 [49].

Among the included studies, eight provided data on the prevalence of MGD in general population samples, while two were conducted in eye-clinic settings. The general population studies were carried out in Singapore, Spain, Russia, Iran, China, and Tehran, whereas the clinical studies involved cataract or eye-clinic patients in Japan and New Zealand. Across all 8 studies, a total of 20,518 adults aged 40 years and older were examined, of whom 18,210 were diagnosed with MGD, reflecting the high burden of disease in this age group. The proportion of women ranged from approximately 51% to 100%, with absolute numbers of female participants between 228 and 2803 per study. Further details on the characteristics of the included studies, including study design, setting, age inclusion criteria, diagnostic methods, and sex distribution, are presented in Table 3.

### 3.2. Risk of Bias Evaluation Results

Quality assessment of the eight included studies was conducted using a modified Newcastle-Ottawa Scale (NOS) adapted for observational cross-sectional designs, with a maximum possible score of 7 points. Two independent reviewers (Z.A. and I.K.) performed parallel quality assessments, with disagreements resolved through consensus with a third reviewer (B.K.).

Overall, six of the eight included studies (75%) achieved adequate methodological quality with NOS scores ≥ 5 points and were retained for the primary meta-analysis. The mean NOS score across all included studies was 5.4 ± 0.9 points (range: 4–6), indicating variable but generally adequate quality. Study-specific quality scores were as follows: Siak, 2012 [7] (Singapore) scored 6 points, reflecting strong participant selection and outcome assessment; Viso, 2012 [25] (Spain) scored 5 points; Amano, 2017 [9] (Japan) scored 6 points; Hashemi, 2017 [5] (Iran) scored 5 points; Hashemi, 2021 [44] (Iran) scored 5 points; Cragi, 2021 [45] (New Zealand) scored 5 points; Bikbov, 2022 [8] (Russia) scored 6 points; and Wang, 2023 [46] (China) scored 4 points.

The most common sources of bias identified across studies were: (1) selection bias: several studies employed convenience or clinic-based sampling rather than truly random population-based sampling, potentially overestimating MGD prevalence; (2) assessment bias: heterogeneous diagnostic criteria ranging from slit-lamp biomicroscopy alone to combined clinical and questionnaire-based approaches, which may have affected comparability of prevalence estimates across studies; and (3) reporting bias: incomplete reporting of demographic characteristics and diagnostic procedures in some studies limited assessment of population representativeness. Notably, all included studies had clear case definitions and documented outcome measurement, which strengthened their methodological rigor. No studies were excluded based on NOS scores, as even the lowest-scoring study (Wang, 2023 [46]; score = 4) contributed valuable data from a distinct geographic region and met all PECOS eligibility criteria. However, the variability in NOS scores and identified sources of bias contributed to the overall low-to-moderate certainty of evidence rating according to GRADE criteria, as detailed in the limitations section.

### 3.3. Meta-Analysis Results

**Figure 2:**
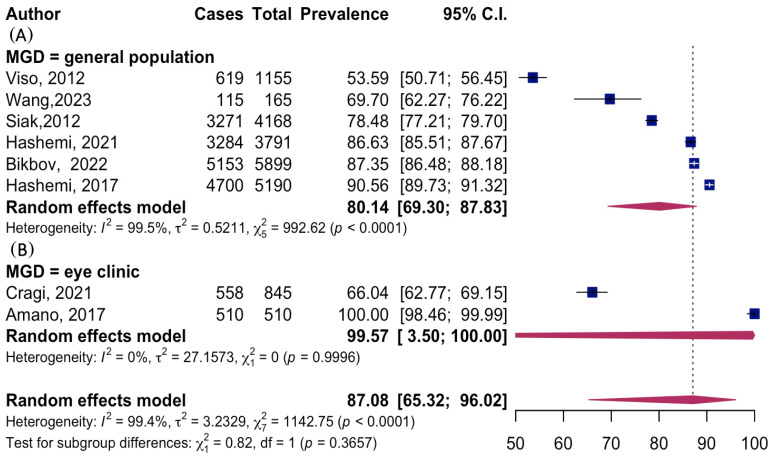
Forest plot of the pooled mean MGD prevalence: (**A**) studies in the general population; (**B**) studies in eye-clinic patients. Group definitions: Viso, 2012 (**A**) [25]—general population; Wang, 2023 (**A**) [46]—general population; Siak, 2012 (**A**) [7]—general population; Hashemi, 2021 (**A**) [44]—general population; Bikbov, 2022 (**A**) [8]—general population; Hashemi, 2017 (**A**) [5]—general population; Cragi, 2021 (**B**) [45]—eye-clinic patients; Amano, 2017 (**B**) [9]—eye-clinic patients.

The mean prevalence of MGD among all included studies was 87.08% (95% CI: 65.32%, 96.02%). The prevalence estimate exhibited high heterogeneity: I^2^ = 99.5%, χ^2^ (df = 7) = 992.62, *p* < 0.0001. When stratified by assessment setting, the mean MGD prevalence estimates were notably lower among studies conducted in the general population: 80.14% (95% CI: 69.30%, 87.83%), though still with high heterogeneity: I^2^ = 99.5%, χ^2^ (df = 5) = 992.62, *p* < 0.0001. Conversely, the mean prevalence estimates were considerably higher in studies that focused on eye-clinic populations: 99.57% (95% CI: 3.50%, 100.00%), also with substantial uncertainty and heterogeneity (I^2^ = 0% likely due to few studies), as presented in Figure 2.

**Figure 3:**
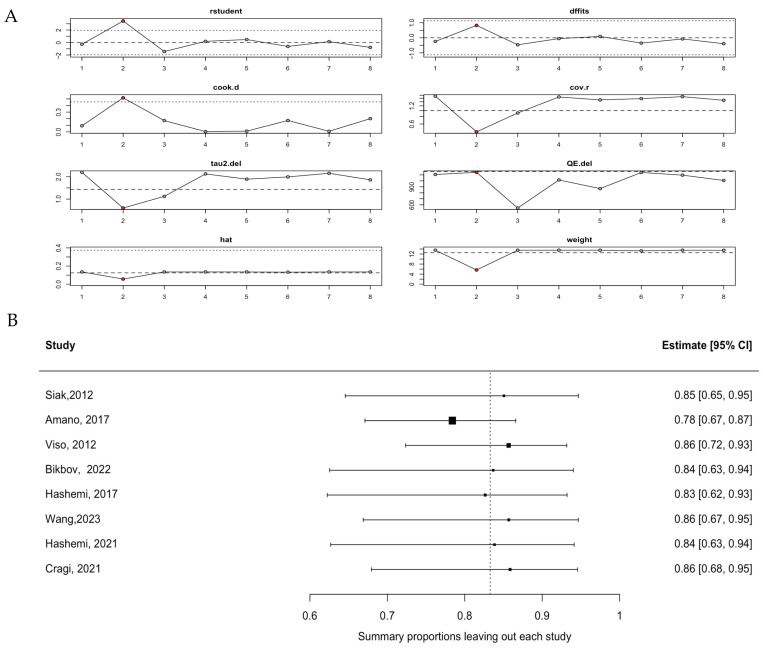
Heterogeneity assessment of the pooled mean MGD prevalence among the general population: (**A**) influence analysis; (**B**) leave one-out analysis for each included study [5,7,8,9,25,44,45,46].

The heterogeneity of the pooled estimate among the general population was further investigated using influence analysis and leave-one-out analysis. Both assessments identified the study by Amano (2017) [9], which reported the highest prevalence (100% in a clinical cataract population), as the most influential study affecting the overall pooled mean prevalence. Its exclusion resulted in a noticeable shift in the summary proportion (pooled prevalence 78% with 95% CI: 67–87%), highlighting its significant impact on the pooled estimate and heterogeneity (Figure 3A,B).

Furthermore, two meta-regression models were constructed to assess the heterogeneity of the pooled MGD prevalence estimate among the general population. The first model evaluated the impact of publication year on the pooled mean prevalence of MGD, while the second model examined the influence of the proportion of female participants. Neither model revealed significant associations at the *p* < 0.05 cutoff point. As shown in the scatter plot, the regression line for the proportion of females is nearly horizontal with a slight downward trend, indicating that the prevalence of MGD does not significantly change as the proportion of women in the study population increases. The data points are widely dispersed, particularly at lower proportions of females, and the presence of a potential outlier at the top of the graph further confirms the lack of a consistent linear relationship (see Supplemental Figures S1 and S2).

### 3.4. Certainty of Evidence Evaluation

The certainty of evidence for the pooled MGD prevalence estimate was assessed using the GRADE (Grading of Recommendations Assessment, Development and Evaluation) framework, which evaluates evidence across five domains: risk of bias, inconsistency, indirectness, imprecision, and publication bias. The assessment began with a baseline level of LOW certainty, as all included studies were observational in design.

Risk of bias (study limitations): Seven of eight included studies (88%) achieved adequate methodological quality with Newcastle-Ottawa Scale scores ≥ 5 points (mean NOS score, 5.2/7). Only one study (Wang, 2023) [46] scored below 5 points. No downgrade was applied for this domain, as the majority of studies demonstrated adequate participant selection, comparability, and outcome assessment.

Inconsistency (heterogeneity): Substantial statistical heterogeneity was observed in the pooled analysis (I^2^ = 99.5%, *p* < 0.001). A downgrade of one level was applied for inconsistency. Although this heterogeneity is partially explainable by differences in diagnostic methods (slit-lamp biomicroscopy alone versus combined with questionnaires), study settings (general population versus clinical samples), geographic regions, and participant age distributions, the magnitude of heterogeneity (I^2^ > 99%) exceeds what can be fully accounted for by these factors. The subgroup analysis by assessment setting partially explained this heterogeneity, with lower prevalence in general population studies (80.14%) compared to clinical eye-clinic populations (99.57%).

Indirectness (applicability): No downgrade was applied for indirectness. All included studies assessed MGD in adults aged ≥40 years using recognized clinical diagnostic methods (slit-lamp biomicroscopy and/or standardized symptom questionnaires), directly addressing the review’s population, exposure, and outcome criteria.

Imprecision (precision of estimates): A downgrade of one level was applied for imprecision. The 95% confidence interval for the pooled prevalence spanned from 65.32% to 96.02% (width of approximately 31 percentage points), indicating substantial imprecision in the pooled estimate. Although the total sample size (*n* = 20,518) is large, the extreme between-study heterogeneity results in a wide confidence interval that precludes precise estimation of the true population prevalence.

Publication bias: Publication bias assessment was not performed, as the number of included studies (n = 8) was below the minimum threshold of 10 studies required for robust funnel plot asymmetry testing and Egger’s regression analysis.

Overall certainty of evidence: The overall certainty of evidence was rated as Very Low (Table 4), indicating limited confidence that the pooled estimate of MGD prevalence in adults aged ≥40 years (87.08%, 95% CI: 65.32–96.02%) reflects the true population prevalence. Starting from a baseline of Low certainty (observational studies), evidence was downgraded by one level for inconsistency (I^2^ = 99.5%) and one level for imprecision (wide 95% CI). The pooled estimate should be interpreted with caution and contextualized by diagnostic approach and study setting. Further high-quality population-based studies employing standardized diagnostic protocols and consistent age-specific reporting are needed to generate more reliable estimates.

## 4. Discussion

This systematic review and meta-analysis revealed a pooled prevalence of MGD of 87% among adults aged 40 years and older, representing a substantially elevated burden of disease in this population. While higher prevalence rates are anticipated in clinical settings compared to general population samples, as individuals with symptomatic conditions are more likely to seek medical attention, the finding that approximately 80% of community-dwelling adults over 40 years may exhibit signs of MGD warrants careful consideration, though this estimate should be interpreted in the context of the extreme heterogeneity and methodological limitations of the included studies [30]. The substantial difference between general population studies (80.14%) and clinic-based studies (99.57%) underscores the importance of stratifying prevalence estimates by study setting. Clinic-based studies inherently overestimate population-level prevalence because they recruit individuals already seeking ophthalmic care, who are more likely to have meibomian gland dysfunction. Accordingly, the general population subgroup estimate of 80.14% is likely more representative of true community-level prevalence among adults aged ≥40 years.

Sensitivity analysis excluding Amano (2017) [9], which reported 100% MGD prevalence in a cataract surgical population, yielded a lower pooled prevalence of 78% (95% CI: 67–87%). This sensitivity-adjusted estimate may represent a more conservative and potentially more generalizable figure for the broader adult population aged ≥40 years.

The magnitude of this prevalence underscores the critical importance of early detection and timely intervention, particularly given the progressive and often irreversible nature of untreated meibomian gland dysfunction and its potential for severe ocular and systemic sequelae [50,51,52].

MGD is not only highly prevalent but also progressive, with untreated disease leading to structural gland damage and sight-threatening ocular surface complications over time [53]. In the context of an aging global population, the cumulative exposure to risk factors such as long-term screen use, polypharmacy, systemic comorbidities, and environmental stressors further amplifies the lifetime burden of MGD-related morbidity [54].

Given the exceptionally high prevalence of MGD in adults over 40 years observed in this meta-analysis, and the fact that this age group is rapidly expanding in many countries, MGD is poised to become an even more significant public health problem in aging societies [50]. As people live longer, they accumulate multiple risk factors that favor the development and progression of MGD, including hormonal changes, increased use of systemic medications (in particular antihypertensives, antidepressants, and antiglaucoma agents), higher burdens of cardiometabolic disease, and prolonged exposure to digital screens and air-conditioned or polluted environments [55]. These observations suggest that MGD may be more prevalent than previously recognized in adults over 40, supporting the need to consider reframing MGD from a predominantly symptomatic clinic-based problem to a highly prevalent, largely underdiagnosed chronic condition of the aging population [56].

While the pooled estimates suggest a high prevalence of MGD among adults aged ≥40 years, these findings should be interpreted with considerable caution given the very low certainty of the evidence (GRADE: Very Low), extreme statistical heterogeneity, heterogeneity in diagnostic criteria across studies, and the mixture of general population and clinic-based study settings. These results should be viewed as hypothesis-generating and underscore the need for large-scale, population-based studies using standardized diagnostic criteria to establish more reliable estimates of MGD prevalence.

Future efforts should therefore prioritize the integration of simple meibomian gland assessment into routine eye examinations for middle-aged and older adults, even in the absence of overt symptoms, with particular attention to individuals with intensive near-work or screen-based occupations, cardiometabolic comorbidities, or long-term topical and systemic medication use [57,58]. At the health-system level, development of risk-stratified screening algorithms, age-adapted patient education materials, and stepwise management pathways—ranging from basic eyelid hygiene and lifestyle modification to targeted pharmacologic and device-based therapies—may help slow disease progression, preserve gland structure, and reduce the long-term burden of MGD-related visual disability in aging populations [59].

## 5. Limitations

This systematic review and meta-analysis has several important limitations that should be acknowledged. First, the pooled prevalence estimate demonstrated substantial heterogeneity (I^2^ = 99.5%, *p* < 0.0001), reflecting considerable methodological variability across included studies in diagnostic criteria (slit-lamp biomicroscopy alone versus combined with questionnaires), population characteristics (age ranges, ethnicity), and assessment settings (community-based versus clinical samples). Second, the relatively small number of eligible studies *(n* = 8) limited the statistical power of subgroup analyses and meta-regression models to fully explain sources of heterogeneity, and precluded robust assessment of publication bias using funnel plot asymmetry tests. Third, the restriction to English-language publications and observational study designs may have introduced language and publication bias, potentially excluding relevant data from non-English literature and limiting causal inference regarding MGD determinants.

Additionally, the search strategy, while utilizing MeSH terms and key epidemiological terms, did not include all possible synonyms (e.g., ‘meibomian gland disease,’ ‘lid margin disease,’ ‘evaporative dry eye’), which may have limited the comprehensiveness of the search. Manual reference checking of included studies and relevant reviews was performed to partially mitigate this limitation.

Geographic representation: The included studies were limited to seven countries across three WHO regions (Western Pacific: Singapore, Japan, China, New Zealand; European: Spain, Russia; Eastern Mediterranean: Iran). Notable gaps exist in epidemiological data from the African Region, Region of the Americas, and South-East Asia Region, which collectively represent more than half of the global population. Consequently, the pooled prevalence estimates may not be fully representative of global MGD burden, and caution should be exercised when generalizing findings to populations in underrepresented geographic regions, particularly those with different genetic backgrounds, environmental exposures, and healthcare access patterns.

Finally, the certainty of evidence was rated as low according to GRADE criteria due to methodological limitations in some included studies (average NOS score variability), inconsistency of effect estimates (high I^2^ values), which collectively warrant cautious interpretation of the pooled prevalence estimates and underscore the need for standardized diagnostic protocols and larger multinational studies in adults aged 40 years and older.

## 6. Conclusions

This systematic review and meta-analysis demonstrates that MGD is frequently identified in adults aged 40 years and older, but reported prevalence varies widely across studies. The substantial heterogeneity observed is largely attributable to differences in diagnostic definitions, assessment methods, and study settings. As a result, a single pooled prevalence estimate should not be interpreted as reflecting the true population prevalence of clinically significant MGD.

Our findings underscore the need for standardized, consensus-based diagnostic criteria and harmonized study designs in future epidemiological research. Population-based studies that distinguish between subclinical gland changes and symptomatic disease are particularly needed to improve clinical interpretability and public health relevance. Until such data are available, estimates of MGD prevalence should be contextualized by diagnostic approach and study population rather than used to infer global disease burden.

## Figures and Tables

**Table 1:** Search strategy used for the systematic review.

Database	Search Criteria	Search Date	Filters
PubMed	Title, Abstract, Keywords	4 November 2025	Language: English
ScienceDirect	Title, Abstract, Author-Specified Keywords	7 November 2025	Language: English Document Type: Research Articles
Scopus	Article title, Abstract, Keywords	11 November 2025	Language: English Document Type: Articles
Web of Science	Title, Abstract, Keywords	15 November 2025	Language: English Document Type: Articles and Early Access
Google Scholar	Title	20 November 2025	Language: English

**Table 2:** Inclusion and exclusion criteria of study selection based on the PECOS framework.

PECOS Framework	Inclusion Criteria	Exclusion Criteria
Population	General adult population aged ≥40 years, including community-based and clinic-based samples.	Studies limited to participants <40 years, pediatric or adolescent samples only, or highly specific experimental populations (animals, in vitro models).
Exposure	Clinically diagnosed MGD identified by slit-lamp biomicroscopy, meibography, lid margin assessment, and/or standardized symptom questionnaires.	Studies assessing only dry eye disease without separate MGD data; studies focusing on the use or effect of specific treatments or devices without reporting MGD prevalence in the target population.
Comparator	Not applicable	Not applicable
Outcome	Number of people diagnosed with MGD out of the total number of participants assessed (proportion or percent).	Studies assessing only treatment efficacy, drug or device toxicity, adverse events, or other outcomes without reporting MGD prevalence.
Study design	Cross-sectional and other observational studies (population-based surveys, cohort baseline assessments, clinic-based observational studies).	Randomized controlled trials, interventional studies, narrative reviews, systematic reviews, editorials, commentaries, conference abstracts, and studies published in languages other than English.

Abbreviations: MGD—meibomian gland dysfunction; PECOS—Population, Exposure, Comparator, Outcome, Study.

**Table 3:** Characteristics of the included studies to the prevalence meta-analysis.

N	Author	Year	Study Design	Country/WHO Region	Age (Range or Mean ±SD)	Number of Population Assessed	# of Population with MGD	Assessment Setting	Assessment Method	Number of Female Participants
1	Siak, 2012 [7]	2012	cross-sectional	Singapore	40–79	4168	3271	General population	Clinical examination and questionnaire administration	1697
2	Viso, 2012 [25]	2012	cross-sectional survey	Spain	40–96	1155	619	General population	Clinical examination and questionnaire administration	390
3	Amano, 2017 [9]	2017	moxed methods	Japan	50–93	510	510	Eye clinic	Clinical examination and questionnaire administration	305
4	Hashemi, 2017 [5]	2017	Cohort Study	Iran	40–64	5190	4700	General population	Clinical examination and questionnaire administration	2769
5	Hashemi, 2021 [44]	2021	cross-sectional study	Iran	68.24 ± 6.53 (60–97)	3791	3284	General population	Clinical examination and questionnaire administration	1889
6	Cragi, 2021 [45]	2021	retrospetive cross-sectional study	New Zealand	45 years old	845	558	Eye clinic	Clinical examination and questionnaire administration	228
7	Bikbov, 2022 [8]	2022	population-based investigation	Russia	40–96	5899	5153	General population	Clinical examination and questionnaire administration	2803
8	Wang, 2023 [46]	2023	prospective study	China	45–60, 60–70 and ≥70 (61.21 ± 8.57)	165	115	General population	Clinical examination and questionnaire administration	115

**Table 4:** GRADE certainty of evidence assessment for prevalence of meibomian gland dysfunction in adults aged ≥40 years.

Domain	Rating	Explanation
Risk of bias (study limitations)	Not serious (0)	7 of 8 studies (88%) achieved adequate quality (NOS ≥ 5); mean NOS score 5.2/7
Inconsistency (heterogeneity)	Serious (1)	Substantial heterogeneity (I^2^ = 99.5%, *p* < 0.001), reflecting variability in diagnostic criteria, population characteristics, and assessment settings
Indirectness (applicability)	Not serious (0)	All included studies assessed MGD in adults aged ≥40 years using slit-lamp biomicroscopy and/or standardized questionnaires, directly addressing the review question
Imprecision (precision)	Serious (−1)	95% CI width ≈ 31 percentage points (65.32–96.02%); total sample size = 20,518 participants across 8 studies
Publication bias	Not assessed	Publication bias assessment not performed due to insufficient number of studies (n = 8; minimum 10 required for funnel plot asymmetry tests)
Overall certainty of evidence	Very Low	Starting level: Low (observational studies). Downgraded 1 level for inconsistency and 1 level for imprecision. Publication bias not assessed (n < 10 studies).

## Data Availability

All data extracted and analyzed in this review are available within the article and its Supplementary Materials. Additional details are available from the corresponding author upon reasonable request.

## Supplementary Materials

The following supporting information can be downloaded at: https://www.mdpi.com/article/10.3390/jcm15083034/s1, Figure S1: Meta-regression bubble plot showing the relationship between publication year and MGD prevalence estimates among general population studies; Figure S2: Meta-regression bubble plot showing the relationship between mean participant age and MGD prevalence estimates among general population studies [8,9,29,30,50,51,52,53]. Table S1: PRISMA Checklist.

## Abbreviations

The following abbreviations are used in this manuscript:

MGD	Meibomian Gland Dysfunction
DED	Dry Eye Disease
GRADE	Grading of Recommendations Assessment, Development and Evaluation
NOS	Newcastle-Ottawa Scale
PRISMA	Preferred Reporting Items for Systematic Reviews and Meta-Analyses
PECOS	Population, Exposure, Comparator, Outcome, Study Design
CI	Confidence Interval
ETDRS	Early Treatment Diabetic Retinopathy Study
WHO	World Health Organization
SD	Standard Deviation
