# Supplementary figures and images for: Systematic Review and Meta-Analysis: Prevalence of Meibomian Gland Dysfunction Among Adults Aged 40 Years and Older
Source: J Clin Med. 2026 Apr 16;15(8):3034. doi: 10.3390/jcm15083034 (PMC13116183; doi:10.3390/jcm15083034)

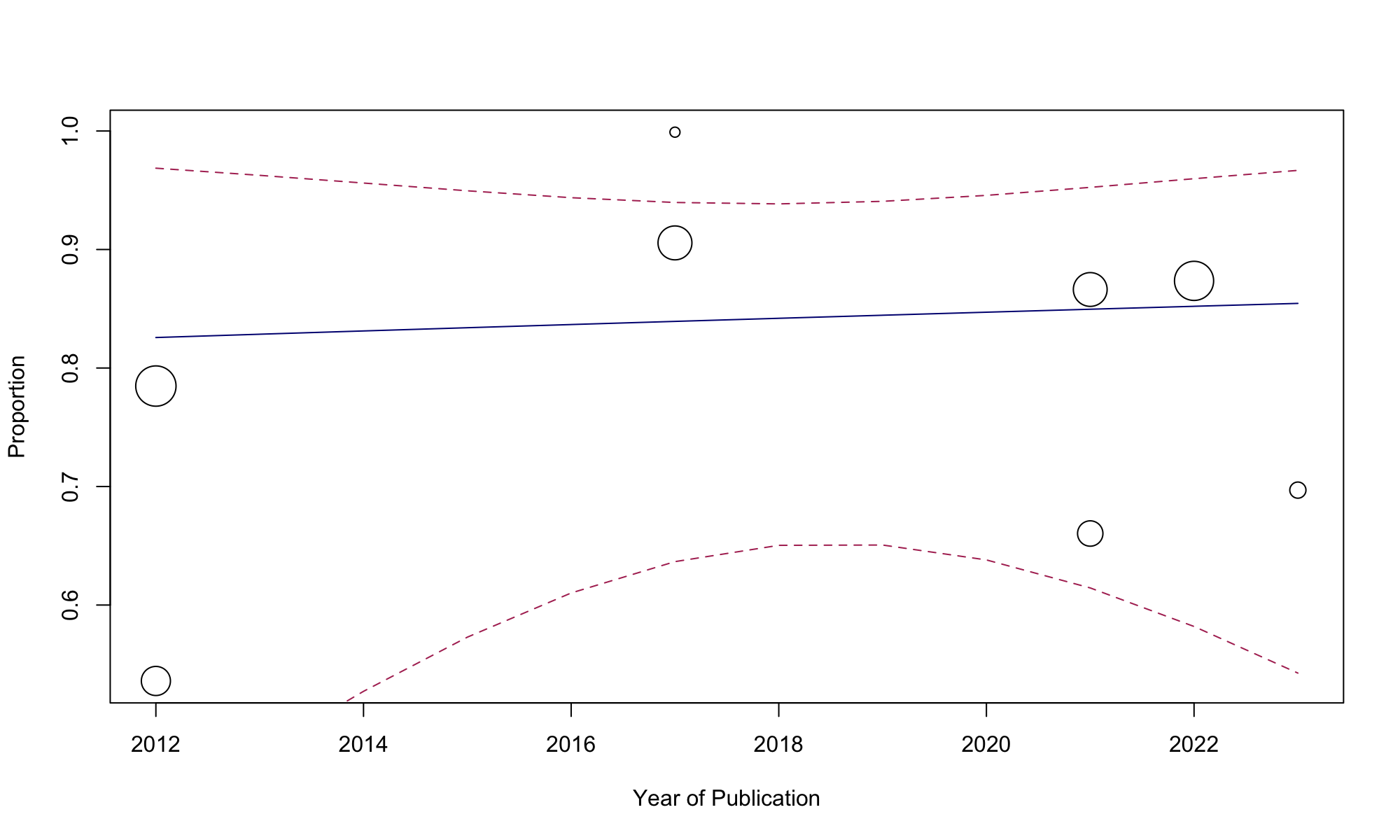

Supplement: Supplementary file 1 [file jcm-15-03034-s001.zip › Figure S1.tiff]

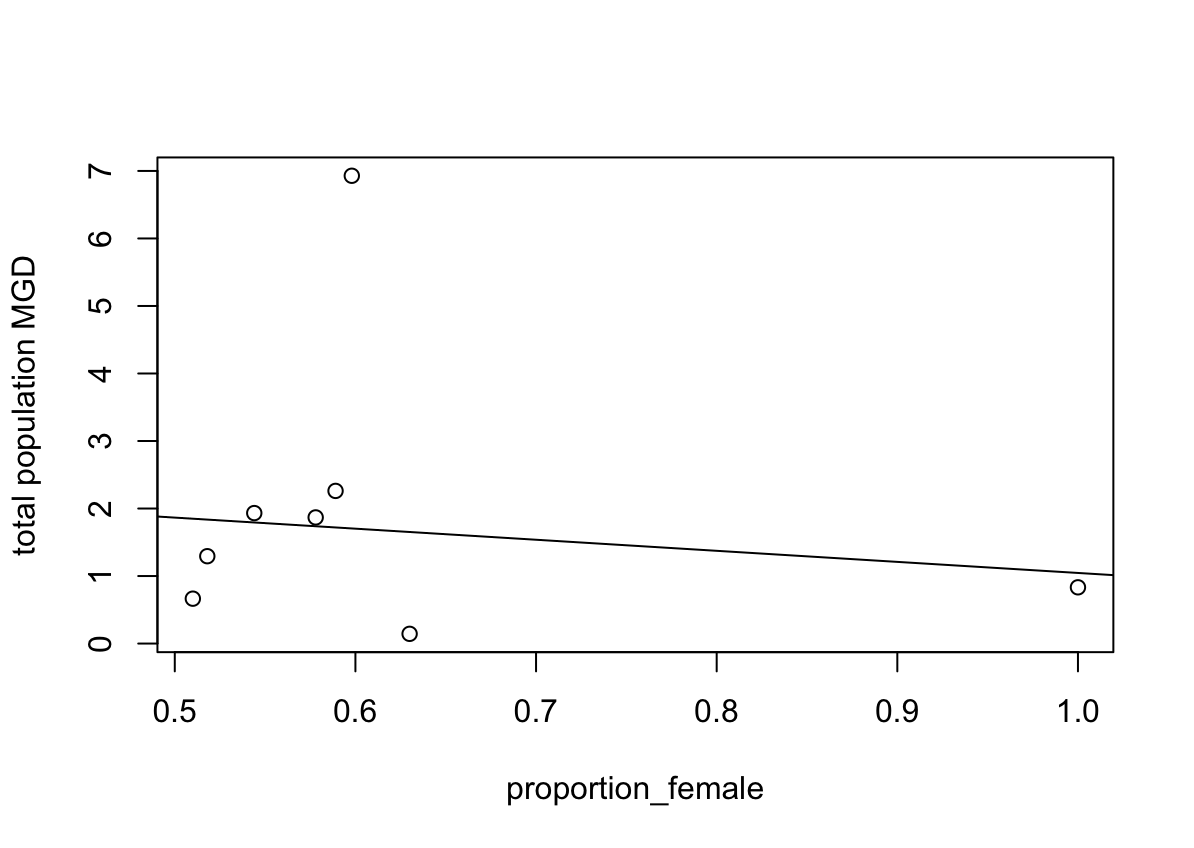

Supplement: Supplementary file 1 [file jcm-15-03034-s001.zip › Figure S2.tiff]
